# Real-World Experiences with Taliglucerase Alfa Home Infusions for Patients with Gaucher Disease: A Global Cohort Study

**DOI:** 10.3390/jcm12185913

**Published:** 2023-09-12

**Authors:** Shoshana Revel-Vilk, Royston Mansfield, Neta Feder-Krengel, Noya Machtiger-Azoulay, David Kuter, Jeff Szer, Hanna Rosenbaum, David Cavalcanti Ferreira, Noa Ruhrman-Shahar, Michael Wajnrajch, Ari Zimran

**Affiliations:** 1 Gaucher Unit, Shaare Zedek Medical Center, Jerusalem 9103102, Israel; azimran@gmail.com; 2 Faculty of Medicine, The Hebrew University of Jerusalem, Ein Kerem, Jerusalem 9112102, Israel; 3 Pfizer R&D UK Ltd., Sandwich, Kent CT13 9NJ, UK; roy.mansfield@pfizer.com; 4 Pfizer, Inc., New York, NY 10001, USA; neta.federkrengel@pfizer.com (N.F.-K.); noya.machtiger-azoulay@pfizer.com (N.M.-A.); michael.wajnrajch@pfizer.com (M.W.); 5 Hematology Division, Massachusetts General Hospital, Harvard Medical School, Boston, MA 02114, USA; dkuter@mgh.harvard.edu; 6 Clinical Haematology at Peter MacCallum Cancer Centre, The Royal Melbourne Hospital, Melbourne 3050, Australia; jeff.szer@mh.org.au; 7 Hematology Day Care and Gaucher Clinic, The Center of Consulted Medicine, Clalit Services, Nazareth 1603701, Israel; roseerlich@gmail.com; 8 Internal Medicine Department, Federal University of Santa Catarina, Florianópolis 88040-900, Brazil; davidcavalcanti1983@gmail.com; 9 Raphael Recanati Genetic Institute, Rabin Medical Center, Beilinson Hospital, Petach Tikva 4941492, Israel; r_noa@hotmail.com; 10 Department of Pediatrics, Grossman School of Medicine, New York University, New York, NY 10016, USA

**Keywords:** adverse event, enzyme replacement therapy, Gaucher disease, home infusion, infusion-related adverse event, safety

## Abstract

Taliglucerase alfa is an enzyme replacement therapy approved for Gaucher disease. We assessed the duration/compliance/safety of such home infusions in commercial use in four countries where home infusion programs are available. The treatment duration/compliance study included 173 patients (Israel, 58; US, 61; Brazil, 48; Australia, 6) who received ≥1 taliglucerase alfa home infusion through 6/2021. The median age at home therapy initiation was 38 (range, 2–87) years; 58% were females. The median treatment duration (at home) was 2.7 (range, 0.04–9.0) years. The annual compliance rate was stable (≥95%) throughout the study period. A search of the Pfizer global safety database (through 6/2021), identified 19 adverse events (AEs) as related to “definite home use” and 14 to “possible home use” of taliglucerase alfa; 42.4% of these AEs were serious; none were fatal. Twelve serious AEs in five separate case reports were considered treatment related: one case of chest discomfort/pain and hypertension and one case of erythema associated with a toe blister, for which causality could not be excluded; pain in extremity; projectile vomiting and chills, alongside excessive eye blinking; and an infusion-related AE (pruritus). In conclusion, this real-life global study demonstrated that taliglucerase alfa home infusions are safe with high compliance rates.

## 1. Introduction

Gaucher disease (GD) is an autosomal recessive lysosomal storage disorder caused by mutations in the *GBA1* gene [1]. The mutations lead to a lysosomal deficiency of glucocerebrosidase activity and consequently to the toxic accumulation of glucocerebroside lipids in various organs (e.g., spleen, liver, bone marrow, and lungs) [2]. Treatments for GD include enzyme replacement therapy (ERT) and substrate reduction therapy (SRT). ERTs provide the enzyme that is deficient/missing and is administered intravenously (IV) every other week, whereas SRTs are oral drugs that reduce the level of the substrate of the deficient/missing enzyme [2]. Three ERTs are currently available. These include imiglucerase (Cerezyme^®^; Genzyme Corporation, Cambridge, MA, US; Food and Drug Administration (FDA) approval, 1994), velaglucerase alfa (VPRIV^®^; Takeda Pharmaceuticals Inc./Shire HGT Inc., Lexington, MA, US; FDA approval, 2010), and taliglucerase alfa (Elelyso^®^; Pfizer, Inc., New York, NY, US; FDA approval, 2012).

Treatment with IV ERT every other week in a hospital setting can adversely impact patients’ quality of life, particularly as this therapy is lifelong. Thus, an option for home infusions is greatly appreciated by patients, as is evidenced by our clinical experience and as was suggested by a UK study that surveyed patients with GD and Fabry disease over a decade ago [3]. In Israel, the US, Brazil, and Australia, home infusions have been an option for patients (following at least three safe infusions in a hospital setting) for years (in some countries since the early 1990s). In other countries, such as Italy, ERT administration has been allowed only at medical centers.

Although all approved ERTs have been successfully administered at home, the literature is limited. The safety of home ERT infusions has been published for alglucerase (the first FDA-approved ERT, which was derived from human placenta and later replaced by ERT produced via recombinant DNA technology) and velaglucerase alfa [4,5,6]. For velaglucerase alfa, the analysis involved 104 adults/children with GD who participated in 1 of 4 velaglucerase alfa studies and demonstrated that the type and severity of infusion-related adverse events (IRAEs) experienced at home and the medical center were similar. Overall, of these 104 patients, 10 (9.6%) experienced IRAEs in a total of 38 infusions. These IRAEs included malaise, pain, hypertension, fatigue, and headache [4]. Another safety analysis focused on Israeli patients (*n* = 174) who received home infusions with velaglucerase alfa in the context of clinical studies, the early access program, and commercial use (a total of 13,845 infusions over nearly nine years) [5]. The study reported no serious adverse events (AEs) with velaglucerase alfa infusions and a compliance rate of 98.4% [5].

Data on the safety of taliglucerase alfa home infusions have not been reported until now, although the overall safety and tolerability of taliglucerase alfa in GD patients are well established [7,8,9,10,11,12]. In the 6 clinical studies investigating the efficacy and safety of taliglucerase alfa in GD, which enrolled a total of 154 patients (adults and pediatric, treatment-naïve and treatment-switched), AEs were generally mild/moderate in severity and transient in nature [12].

For GD patients treated with taliglucerase alfa, Pfizer has been offering home therapy service in Israel (through Pfizer since 2016, before that through Protalix), US (from 2012), Brazil (from Protalix since 2020), and Australia (from 2016). The service is provided by trained nurses either as an in-house service (Israel) or through a third-party vendor (US, Brazil, and Australia). The goal of the current study was to assess the duration, compliance, and safety of home infusions with taliglucerase alfa in commercial use in Israel, the US, Brazil, and Australia.

## 2. Results

### 2.1. Study Patients

This study on treatment duration and compliance included a total of 173 patients (58 from Israel, 61 from the US, 48 from Brazil, and 6 from Australia) who received ≥1 home infusion of taliglucerase alfa between the initiation of taliglucerase alfa home infusion program through Pfizer (except for Brazil, where it is managed through Protalix) and 30 June 2021. Overall, the cohort included more female than male patients (57.8% vs. 42.2%), although the female-to-male ratio varied between the countries included in the analysis (Table 1). The median (range) age at the time of home therapy initiation for the entire cohort was 38 (2–87) years and was similar in Israel, and the US, younger in Brazil, and older in Australia where at the time of this study, there were no pediatric patients in the home infusion program, although such patients are eligible for it (Table 1). Approximately two-thirds (67.6%) of all patients in the cohort were <50 years old; the age distribution was overall similar between Israel and the US with 55.2% and 60.7% of the patients < 50 years, respectively, whereas almost all the study patients from Brazil (93.8%) were <50 years (Table 1).

### 2.2. Treatment Duration and Compliance

As patients may have received taliglucerase alfa in the hospital setting before joining the home infusion program, we examined the total duration of taliglucerase alfa treatment as well as treatment through the home infusion program. Overall, more than half of all patients (52.0%) in the cohort received taliglucerase alfa infusions for a total of ≥5 years, with more than a third of patients (40.5%) receiving taliglucerase alfa for a total of ≥7 years. The majority of the patients in Israel (62.1%), over a third of the patients in the US (36.1%), and a quarter of the patients in Brazil (25.0%) received taliglucerase alfa for a total of ≥7 years (Table 2).

**Figure 1:**
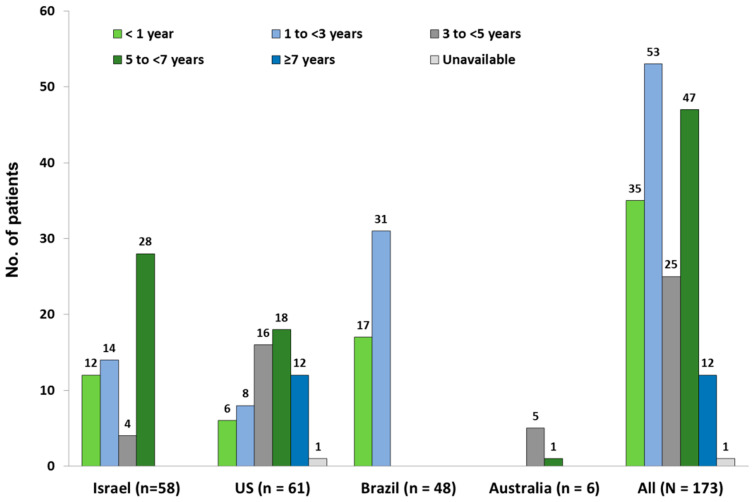
Distribution of the duration of taliglucerase alfa treatment through the home infusion treatment by country.

The median (range) duration of taliglucerase alfa treatment through the home infusion program for all patients in the cohort was 2.7 (0.04–9.0) years. In the US and Israel, the median (range) treatment duration was the longest (US: 4.9 [0.04–9.0] years; Israel: 4.8 [0.04–5.3] years), followed by Australia (4.5 [4.3–5.0] years), and Brazil (1.1 [0.2–1.2] years). The distribution of the duration of taliglucerase alfa home infusion treatment is presented in Figure 1. Overall, 84 patients (48.6%) received home infusions of taliglucerase alfa for ≥3 years. In the US, 46 patients (75.4%) received home infusions of taliglucerase alfa for ≥3 years; in Israel, 32 patients (55.2%) received such infusions for ≥3 years; and in Australia, all 6 patients received such infusions for ≥3 years. In Brazil, however, all patients received taliglucerase alfa home infusions for <3 years.

The calculated number of infusions included in our study, assuming that each patient received an infusion every other week, is 13,952 (Israel, 4526; US, 7479; Brazil, 1225; and Australia, 722). The actual number of infusions administered in Israel was 4361 (96.4% compliance); in Brazil, 1200 (98.0% compliance); and in Australia, 699 (96.8% compliance). The actual number of infusions administered in the US was unavailable. Except for the first several months after the initiation of the home infusion program in Australia, where the compliance was relatively low (61.5%), the annual compliance rate was very high and stable (≥95%) throughout the study period (2016 through H1 2021), including during the COVID-19 pandemic.

### 2.3. Safety

The safety analysis included all taliglucerase alfa AEs in the global safety database (through June 2021). The search of this database identified a total of 163 cases with AEs and the word “home” in the case narrative, of which 130 were excluded from further analysis. The excluded cases included 37 cases where taliglucerase alfa was administered at home in a clinical trial setting, 80 cases with no evidence of home infusion, 2 cases in countries with no organized home therapy program (Chile, Serbia), and 11 cases with evidence of home administration, but no symptomatic AEs (product dose omission, product storage error, product administration error, or inappropriate schedule of product administration).

The 33 remaining cases were classified as either “definite” (*n* = 19) or “possible” (*n* = 14) home use. The characteristics of these 33 cases are presented in Table 3. Overall, 14 cases were considered serious and none were fatal. Over half of all safety cases (18 patients) involved female patients, and only 1 case involved a pediatric patient. Cases were reported in all countries with home infusion programs (Israel, the US, Australia, and Brazil). No cases with symptomatic AEs indicated self-administration by the patient or with the help of a non-medical caregiver, although 10 case reports did not explicitly mention the presence of a healthcare professional (e.g., a nurse or physician) at the time of the infusion.

The most frequently reported AE-preferred terms (MedDRA) of these 33 cases by causality and seriousness are presented in Table 4. Some of the AEs are recognized as adverse reactions for taliglucerase alfa (i.e., listed in the product reference information [13]). These include fatigue, pain in extremities, bone pain, arthralgia, infusion-related reaction, pruritus, and chest discomfort as well as headache, weight increase, abdominal pain, dizziness, erythema, flushing, and rash. The blood pressure increase is currently not listed as an adverse drug reaction. However, the reported hypertension appeared to be associated with underlying comorbidities and the temporal relationship with taliglucerase alfa is unclear; a causal relationship cannot be completely excluded.

Additionally, events such as chest pain, asthenia, nasal congestion, rhinorrhoea, and somnolence are considered adverse reactions often associated with IRAEs, whereas other AEs, including malaise, nasopharyngitis, feeling abnormal, pain, cataract, chapped lips, COVID-19, influenza, influenza-like illness, rhinorrhoea, and urinary tract infection, were considered intercurrent conditions unrelated to taliglucerase alfa treatment. Lastly, bone disorder was considered to be associated with the underlying GD.

Twelve serious AEs in 5 separate cases were considered to be treatment related. The first case involved pain in the extremities (difficulty in bending fingers, for which surgery was carried out) and was causally attributed to taliglucerase alfa, although the underlying GD could be an alternative explanation. In the second case, home care staff reported that an hour after starting an infusion, the patient had itching in the lower limbs (pruritus), which was regarded as an IRAE. The symptoms improved after administration of 0.9% saline and hydrocortisone and the infusion was completed without further complications. The third case involved serious events of chest discomfort, chest pain, and hypertension (accompanied by atrial fibrillation), which had limited information to support drug causality. However, since it occurred during treatment, causality cannot be excluded. The fourth case reported erythema associated with a blister on the toe, which was noted during an infusion by the attending nurse, and due to the temporal association, causality cannot be excluded. The fifth case reported serious projectile vomiting, chills, and excessive eye blinking during infusion in a three-year-old patient who required treatment with epinephrine. The case also reported serious growth retardation, although this is likely a result of the underlying GD.

Nine cases reported serious events that were considered not to be related to treatment with taliglucerase alfa. The events reported in these cases included those associated with the underlying GD such as bone disorders, blood iron decrease, cholecystitis, and cholelithiasis. Other unrelated events were associated with intercurrent medical conditions including infections such as COVID-19, pneumonia and cystitis, syncope and falls with associated limb fractures, and events such as cataracts, vomiting and intestinal blockage, thrombosis, nephrolithiasis and asthma.

## 3. Discussion

The current analysis of the global safety database of Pfizer and the aggregated data from the four countries where the home infusion program is currently available demonstrates that home infusions of taliglucerase alfa are feasible, safe, and associated with high compliance rates. The safety profile in the home setting is consistent with that observed in the clinical setting, and with the profile of the patient population (i.e., more AEs were reported in females vs. males and only one case was reported in children). No specific or enhanced risk with home administration was noted (all AEs with causal associations are well characterized as AEs for taliglucerase alfa or as IRAEs).

Our finding that taliglucerase alfa can be administered safely in the home setting is consistent with prior studies examining ERT [1,4,5,6]. Notably, as home infusions are offered to patients with at least three initial uneventful infusions of taliglucerase alfa in a hospital setting, the population of patients receiving home infusions is enriched in patients who tolerate the drug well. Home infusions offer advantages such as patient convenience, improved quality of life, and high compliance [14]. In a recent survey of 125 patients receiving home infusions in Argentina for lysosomal diseases including GD (13% of patients), 88.5% of the respondents considered their quality of life improved, and the most valuable aspect of home infusion (noted by 77% of the respondents) was that the patients felt comfortable receiving the infusion in their own home. Other advantages noted by more than half of the respondents included the ability to adjust the infusion to their daily routine, timetable flexibility, and that the nurse had more time to dedicate to patient care [15]. In this study, the nurses providing the care were also surveyed. From their perspective, for 96% of the patients, quality of life improved. The adherence rate (across all diseases) was 93%, which is consistent with that observed in the current GD-exclusive study [15]. Also, although not evaluated for ERT specifically, home infusions, in general, have been shown to be associated with cost savings [14].

The advantages of a home infusion program are underscored during a pandemic, such as the recent SARS-CoV-2 pandemic, since home infusions reduce patients’ infection risk due to reduced exposure. A recent study analyzed the impact of the SARS-CoV-2 pandemic in the Spanish GD community through a survey of 110 patients. Of these 110 patients, 40% received hospital-based ERT and 6% received home-based ERT. The remaining patients received SRT (45%) or no therapy (9%) [16]. Interestingly, 25% of the patients treated with hospital-based ERT reported therapy interruptions, and 50% of all patients (regardless of treatment) reported being worried about their predisposition to a severe SARS-CoV-2 infection [16]. Therefore, specifically during a pandemic, a home infusion program could promote continuity of care and improve quality of life by reducing patients’ anxiety regarding their risk [16]. Indeed, our findings demonstrate that the compliance rate remained high, even during the COVID-19 pandemic period (2020 through H1 of 2021). Although interesting, health-related quality of life was not captured in this study.

The strengths of our study include its true representation of real-world data (data from four countries in different areas of the world and with different healthcare systems). The limitations of our study stem from its design and the reliability of post-marketing AE reporting that is, in general, submitted voluntarily (except for healthcare professionals who administer treatment and are contracted through Pfizer, as those are required to report any AEs they observe). Also, the data had a relatively large number of cases (80) that were excluded due to a lack of clear evidence of home infusion, and AEs in-home use could have been missed if the word “home” did not appear in the case narrative. As the magnitude of underreporting is unknown, incidence rates could not be calculated and between-drug comparisons could not be made. Also, due to the study design, causality cannot be established, as the AE may have been caused by the underlying disease, or concomitant medication, for example.

## 4. Materials and Methods

For analyses involving patient demographics and drug administration, aggregated data were provided from Israel, the US, Brazil, and Australia from the initiation of home infusion therapy through Pfizer (or Protalix for Brazil), which was 2012 for the US, 2016 for Israel and Australia, and 2020 for Brazil, and through 30 June 2021. The eligibility criteria for these analyses included patients who were in the home infusion program (not in a clinical trial setting) up to 30 June 2021, and who received at least one taliglucerase alfa infusion at home. Patients who were in the program, but did not undergo infusion at home during this timeframe were excluded.

For the safety analysis, the study entailed a cumulative search of all taliglucerase alfa AE cases in the Pfizer global safety database through 30 June 2021 where the word “home” was included in the case narrative. The Pfizer global safety database contains anonymized AE reports from patients, healthcare professionals, registries, licensing partners, and the literature reports of serious and non-serious AEs for the company’s products. In addition, the database contains serious AE reports from investigational studies conducted by Pfizer Research and Development, Pfizer Business Units, and from non-research post-marketing studies, regardless of causality. For patients in the taliglucerase alfa home infusion program, AEs are typically reported by the nurses who administer the treatment. The nurses are required to report all AEs that they observe or those reported by the patient.

AE cases where the word “home” was identified were individually reviewed and classified as definite home use, possible home use (i.e., suggestive but not definitive information to indicate that taliglucerase alfa was received in a home setting), unknown, and not relevant (i.e., use in a clinical trial setting). Cases that did not involve a symptomatic AE (i.e., product dose omission, product storage error, product administration error, or inappropriate schedule of product administration) were excluded. AE cases that were classified as definite or possible home use were then categorized according to the causal association between the AE and taliglucerase alfa infusion (as determined by either the reporting healthcare practitioner or when the AE was entered into the Pfizer system by the supervising medical officer). Serious cases were defined as those leading to death, hospitalization, prolongation of hospitalization, disability, are life-threatening, cause congenital abnormalities, or require medical intervention to prevent any of the former. Non-serious cases were defined as all other cases.

Descriptive statistics were used to summarize the characteristics of the study population using frequencies with percentages for categorical variables and medians with ranges for continuous variables.

The study was exempt from institutional review board (IRB) approval because all patients provided written informed consent for data collection and since the data provided for the analysis were received from Pfizer’s databases in an aggregated and anonymized form.

## 5. Conclusions

This real-life global cohort study involving a total of 173 patients from Israel, the US, Australia, and Brazil, who received a total of over 10,000 home infusions of taliglucerase alfa over four years, demonstrates that such infusions are feasible, safe, and associated with high compliance rates. During a global health crisis such as the recent COVID-19 pandemic, a home infusion option is crucial for safe and uninterrupted medical care for GD patients on ERT.

## Figures and Tables

**Table 1:** Patient demographics.

	Israel *n* = 58	US *n* = 61	Brazil *n* = 48	Australia *n* = 6	All *n* = 173
Sex, *n* (%)					
Female	26 (44.8)	40 (65.6)	29 (60.4)	5 (83.3)	100 (57.8)
Male	32 (55.2)	21 (34.4)	19 (39.6)	1 (16.7)	73 (42.2)
Age at the time of home therapy initiation, years					
Median (range)	44 (2–87)	45 (3–81) *	25 (8–60)	52 (25–72)	38 (2–87)
Age category, years, *n* (%)					
<18	3 (5.2)	3 (4.9)	11 (22.9)	0 (0)	17 (9.8)
18–29	7 (12.1)	8 (13.1)	17 (35.4)	1 (16.7)	33 (19.1)
30–39	14 (24.1)	12 (19.7)	15 (31.3)	1 (16.7)	42 (24.3)
40–49	8 (13.8)	14 (23.0)	2 (4.2)	1 (16.7)	25 (14.5)
50–59	9 (15.5)	10 (16.4)	2 (4.2)	1 (16.7)	22 (12.7)
60–69	11 (19.0)	10 (16.4)	1 (2.1)	1 (16.7)	23 (13.3)
≥70	6 (10.3)	3 (4.9)	0 (0)	1 (16.7)	10 (5.8)
Unavailable	0 (0)	1 (1.6)	0 (0)	0 (0)	1 (0.6)

* Age information was unavailable for one patient.

**Table 2:** Duration of total treatment with taliglucerase alfa.

	Israel *n* = 58	US *n* = 61	Brazil *n* = 48	Australia *n* = 6	All *n* = 173
Duration category, years, *n* (%)					
<1	8 (13.8)	3 (4.9)	4 (8.3)	0 (0)	15 (8.7)
1–<3	6 (10.3)	8 (13.1)	10 (20.8)	0 (0)	24 (13.9)
3–<5	7 (12.1)	11 (18.0)	19 (39.6)	5 (83.3)	42 (24.3)
5–<7	1 (1.7)	16 (26.2)	2 (4.2)	1 (16.7)	20 (11.6)
≥7	36 (62.1)	22 (36.1)	12 (25.0)	0 (0)	70 (40.5)
Unavailable	0 (0)	1 (1.6)	1 (2.1)	0 (0)	2 (1.2)

**Table 3:** Overview of safety cases for taliglucerase alfa in-home use.

Parameter	Total No. of Cases *n* = 33
**Sex, *n* (%)**	
Female	18 (54.5)
**Age category, years, *n* (%)**	
≤17	1 (3.0)
18–30	5 (15.2)
31–50	11 (33.3)
51–64	10 (30.3)
65–74	3 (9.1)
Unknown	3 (9.1)
**Fatal cases, *n* (%)**	0 (0)
Case seriousness, * *n* (%)	
Serious	14 (42.4)
Nonserious	19 (57.6)
**Country, *n* (%)**	
Israel	14 (42.4)
US	11 (33.3)
Australia	4 (12.1)
Brazil	4 (12.1)

* Serious cases were defined as those reporting adverse events leading to death, hospitalization, prolongation of hospitalization, disability, are life-threatening, cause congenital abnormalities, or require medical intervention to prevent any of the former; non-serious cases were defined as all others.

**Table 4:** Symptomatic AEs in patients with definite/possible home use of taliglucerase alfa by system organ class and frequency (reported at least twice in the total case reports), association with treatment and seriousness.

System Organ Class and Preferred Term	No. of Case Reports		
	Total	All Treatment-Related Events	Treatment-Related Serious Events
**General disorders and administration site conditions**			
Fatigue	6	2	0
Malaise	5	0	0
Chest discomfort	4	4	1
Chest pain	4	3	1
Feeling abnormal	3	0	0
Pain	3	2	0
Asthenia	2	1	0
Influenza-like illness	2	0	0
**Musculoskeletal and connective tissue disorders**			
Pain in extremities	6	2	1
Bone pain	6	1	0
Arthralgia	5	1	0
Bone disorder	3	0	0
**Infections and infestations**			
Nasopharyngitis	5	0	0
COVID-19	2	0	0
Influenza	2	0	0
Urinary tract infection	2	1	0
**Skin and subcutaneous tissue disorders**			
Pruritus	6	4	1
Erythema	2	2	1
Rash	2	2	0
**Injury, poisoning, and procedural complications**			
Infusion-related reaction	5	5	1
Fall	3	0	0
**Respiratory, thoracic, and mediastinal disorders**			
Cough	2	0	0
Nasal congestion	2	2	0
Oropharyngeal pain	2	0	0
Rhinorrhoea	2	1	0
**Nervous system disorders**			
Headache (head discomfort)	3	1	0
Dizziness	2	1	0
Somnolence	2	1	0
**Gastrointestinal disorders**			
Abdominal pain	2	0	0
Chapped lips	2	1	0
Gastric disorder	2	0	0
**Investigations**			
Weight increased	3	2	0
Blood pressure increased	2	2	0
**Vascular disorders**			
Flushing	2	2	0
Hypertension	2	2	1
**Eye disorders**			
Cataract	2	0	0

## Data Availability

The data presented in this study are available on request from the corresponding author.
